# Seed Extract of *Psoralea corylifolia* and Its Constituent Bakuchiol Impairs AHL-Based Quorum Sensing and Biofilm Formation in Food- and Human-Related Pathogens

**DOI:** 10.3389/fcimb.2018.00351

**Published:** 2018-10-25

**Authors:** Fohad Mabood Husain, Iqbal Ahmad, Faez Iqbal Khan, Nasser A. Al-Shabib, Mohammad Hassan Baig, Afzal Hussain, Md Tabish Rehman, Mohamed F. Alajmi, Kevin A. Lobb

**Affiliations:** ^1^Department of Food Science and Nutrition, College of Food and Agriculture Sciences, King Saud University, Riyadh, Saudi Arabia; ^2^Department of Agricultural Microbiology, Aligarh Muslim University, Aligarh, India; ^3^Department of Chemistry, Rhodes University, Grahamstown, South Africa; ^4^School of Biotechnology, Yeungnam University, Gyeongsan, South Korea; ^5^Department of Pharmacognosy, College of Pharmacy, King Saud University, Riyadh, Saudi Arabia

**Keywords:** *Psoralea corylifolia*, bakuchiol, quorum sensing, biofilm, molecular dynamics simulation

## Abstract

The emergence of multi-drug resistance in pathogenic bacteria in clinical settings as well as food-borne infections has become a serious health concern. The problem of drug resistance necessitates the need for alternative novel therapeutic strategies to combat this menace. One such approach is targeting the quorum-sensing (QS) controlled virulence and biofilm formation. In this study, we first screened different fractions of *Psoralea corylifolia* (seed) for their anti-QS property in the *Chromobacterium violaceum* 12472 strain. The methanol fraction was found to be the most active fraction and was selected for further bioassays. At sub-inhibitory concentrations, the *P. corylifolia* methanol fraction (PCMF) reduced QS-regulated virulence functions in *C. violaceum* CVO26 (violacein); *Pseudomonas aeruginosa* (elastase, protease, pyocyanin, chitinase, exopolysaccharides (EPS), and swarming motility), *A. hydrophila* (protease, EPS), and *Serratia marcescens* (prodigiosin). Biofilm formation in all the test pathogens was reduced significantly (*p* ≤ 0.005) in a concentration-dependent manner. The β-galactosidase assay showed that the PCMF at 1,000 μg/ml downregulated *las*-controlled transcription in PAO1. *In vivo* studies with *C. elegans* demonstrated increased survival of the nematodes after treatment with the PCMF. Bakuchiol, a phytoconstituent of the extract, demonstrated significant inhibition of QS-regulated violacein production in *C. violaceum* and impaired biofilm formation in the test pathogens. The molecular docking results suggested that bakuchiol efficiently binds to the active pockets of LasR and RhlR, and the complexes were stabilized by several hydrophobic interactions. Additionally, the molecular dynamics simulation of LasR, LasR–bakuchiol, RhlR, and RhlR–bakuchiol complexes for 50 ns revealed that the binding of bakuchiol to LasR and RhlR was fairly stable. The study highlights the anti-infective potential of the PCMF and bakuchiol instead of bactericidal or bacteriostatic action, as the extract targets QS-controlled virulence and the biofilm.

## Introduction

Quorum sensing (QS) is a density-dependent phenomenon facilitating the coordinated regulation of gene expression in bacteria (Winans and Bassler, 2002). N-acyl homoserine lactone (AHL) based QS systems in gram-negative bacteria are the most studied (Wu et al., 2004). With increasing population densities, AHL levels increase and reach threshold concentrations that allow binding to specific regulators, and the resulting complexes then regulate the expression of various genes (Papenfort and Bassler, 2016). Various food- and human-related pathogens employ QS to regulate genes that code for virulence, production of secondary metabolites, plasmid transfer, motility, and biofilm formation (Williams, 2007; Whiteley et al., 2017). Since QS controls virulence, pathogenicity, and biofilm formation, interfering with QS offers an alternative therapeutic strategy that targets the functions that are not essential for the survival of the bacteria and therefore are subject to less selective pressures as observed for conventional drugs (Bjarnsholt and Givskov, 2008; Lowery et al., 2010). Interfering with the bacterial communication forces the bacteria to reside as individuals fending for themselves, whereas the bacteria residing and functioning as a group build strong defense that an individual bacterium finds impossible to achieve (Rasmussen and Givskov, 2006). This strategy of targeting the functions of bacteria that are responsible for pathogenesis rather than growth have been termed as “antivirulence” or “antipathogenesis” therapies (LaSarre and Federle, 2013; de la Fuente-Núñez et al., 2014).

The first QS inhibitory activity was determined in furanones isolated from *Delisea pulchra*, a seaweed (Rasmussen et al., 2000). Numerous QS inhibitors (QSIs) have been reported since the discovery of furanones, and few have been tested in animal models with great success. Unfortunately, studies showed that these compounds are unstable and toxic, and hence, unsuitable for human use (Rasmussen and Givskov, 2006). Therefore, there is an urgent need to search for other safe and stable anti-QS agents.

The use of medicinal plants has increased considerably in the last decade or so, with an estimated 80% of the populations mostly from developing countries relying on traditional medicines for their primary health care (Ahmad et al., 2006; WHO, 2011, 2012). Recently, an increased interest has been shown by the scientific community to screen and search anti-QS activity from natural products (Husain and Ahmad, 2013; Kalia, 2013; Reen et al., 2018). QS inhibitors have also been reported in various natural products including extracts of medicinal plants (Adonizio et al., 2006, 2008a; Omwenga et al., 2017), fruits and spices (Huerta et al., 2008; Abraham et al., 2012; Husain et al., 2015a, 2017), and phytocompounds (Vandeputte et al., 2010, 2011; Husain et al., 2015b; Al-Yousef et al., 2017; Musthafa et al., 2017).

*Psoralea corylifolia* (Fabaceae) is an annual herb that is widely used both in Ayurvedic as well as in Chinese traditional medicine as a cardiac tonic, vasodilator, and pigment and has antitumor, antibacterial, cytotoxic, and anthelminthic effects. The seeds of *P. corylifolia* are used for its laxative, aphrodisiac, anthelminthic, diuretic, and diaphoretic effects for febrile patients in the traditional system of medicine (Chopra et al., 2013).

Keeping in mind the medicinal properties of *P. corylifolia*, in the present investigation, we screened different fractions of *P. corylifolia* (seed) for their QS inhibition in *Chromobacterium violaceum*. The most active fraction and its major phytoconstituent were selected for further studies on QS-controlled virulence and biofilm formation in various food- and human-related pathogens.

## Materials and methods

### Bacterial strains

The bacterial strains under study were *Pseudomonas aeruginosa* PAO1, *P. aeruginosa* PAF79, *C. violaceum* ATCC 12472, *C. violaceum* CVO26, Aeromonas hydrophila WAF38, *Serratia marcescens*, and Listeria monocytogenes (laboratory strains). All strains were maintained on the Luria Bertani (LB) broth solidified with 1.5% agar (Oxoid).

### Collection of plant material and extraction

*Psoralea corylifolia* (PC) seeds were obtained from The Himalaya Drug Company, Dehradun (Uttarakhand). Seeds of PC were ground to powder and extracted sequentially by the method described by Husain et al. (2015a). First, the petroleum ether fraction was dried using a rotary evaporator at 40°C followed by successive sequential extraction with other solvents (benzene, ethyl acetate, acetone, and methanol). Each of the dried fraction was collected and stored at 4°C and reconstituted in DMSO (0.1%) for experimental use.

### Screening of fractions for quorum sensing inhibition

The standard method of McLean et al. (2004) was adopted to screen *P. corylifolia* for anti-QS activity. LB agar plates were overlaid with 5 ml LB soft agar containing 10^6^ CFU/ml of *C. violaceum* ATCC 12472. Wells of 8 mm size were punched and sealed with 1–2 drops of molten agar (0.8% agar). The wells were loaded with different concentrations of 100 μl of plant extract. A solvent blank was used as the negative control. The inhibition of purple pigmentation in *C. violaceum* ATCC 12472 around the disk impregnated with the extract was considered as positive anti-QS.

### Determination of minimum inhibitory concentration (MIC)

The minimum inhibitory concentration (MIC) of the PC seed extract against test bacteria was determined by using the micro broth dilution method, described by Eloff (1998).

### Effect of sub-MICS of methanol fraction on violacein production in *chromobacterium violaceum* CVO26

Overnight-grown *C. violaceum* CV026 (OD_600nm_ = 0.1) was inoculated to Erlenmeyer flasks containing LB, LB supplemented with C6-HSL (10 μM/l), and LB supplemented with C6-HSL and sub-MICs of the extract. The flasks containing treated and untreated CVO26 were incubated at 27°C with 150 rev/min agitation for 24 h (Choo et al., 2006). The effect of the seed extract on violacein production in *C. violaceum* (CVO26) was determined using the method of Blosser and Gray (2000).

### Effect of sub-MICS of methanol fraction on QS-regulated virulence

The sub-MICs of the methanol fraction of *P. corylifolia* (seed) were used to study the QS-regulated virulence functions in *P. aeruginosa* [LasB, pyocyanin, protease, chitinase, swarming motility, and exopolysaccharide (EPS) production], *A. hydrophila* (protease and EPS production), and *S. marcescens* (prodigiosin). The method of Husain et al. (2015a) was adopted to study the virulence functions in *P. aeruginosa* and *A. hydrophila*, while the determination of prodigiosin was performed by adopting the protocol described by Morohoshi et al. (2007).

### Assay for biofilm inhibition

The effect of the sub-MICs of the PCMF on biofilm formation was studied using the microtiter plate (MTP) assay (O'Toole and Kolter, 1998). Briefly, overnight-grown test bacteria were resuspended in a fresh LB medium in the presence and the absence of sub-MICs of the PCMF and incubated at 30°C for 24 h. The biofilm inhibition in the MTP was determined by crystal violet staining and measuring the absorbance at OD_470nm_.

### β-Galactosidase assay

The β-galactosidase reporter activity was assayed as described by Husain et al. (2015b). Briefly, a supernatant of overnight cultures of PAO1 grown in the presence and absence of the sub-MICs of the PCMF was extracted with ethyl acetate for AHLs. Then, 0.5 ml of the extracted supernatant and 2 ml of the *E. coli* MG4 (pKDT17) (Zhou et al., 2013) strain were incubated at 30°C in a water bath rotating at 100 rpm for 5 h. The cells were centrifuged (3,200 g for 15 min) and the resultant cell pellet was suspended in an equal volume of the Z-buffer (Na_2_HPO_4_.7H_2_O, 0.06 M; NaH_2_PO_4_.H_2_O, 0.04 M; KCl, 0.01 M; MgSO_4_.7H_2_O, 0.001 M; β-mercaptoethanol, 0.05 M; pH 7.0). To lyse the cells, 1 ml of cell suspension, 1ml of the Z-buffer, 200 μl of chloroform, and 100 μl of 0.1% sodium dodecyl sulfate were added; further, 0.4 ml of O-nitrophenol-β-D-galactopyranoside was also added. To stop the reaction after the development of yellow color, 1 ml of 1 M Na_2_CO_3_ was used. Optical density (OD) was measured at 420 and 550 nm. The units of β-galactosidase were calculated as 1,000 × OD_420nm_-(1.75 × OD_550nm_)/time × volume × OD_600nm_.

### Caenorhabditis elegans survival assay

The method described by Musthafa et al. (2012) was adopted to study the antipathogenic potential of the PCMF *in vivo* in the *C. elegans* nematode model. Briefly, PAO1-infected nematodes were incubated at 25°C for 12 h. Incubated *C. elegans* were washed thrice with the M9 buffer to remove surface-bound bacteria. Approximately ten PAO1-infected worms were transferred to the wells of the MTP containing the PCMF treatment/untreated 10% LB broth in the M9 buffer and incubated at 25°C. Every 12 h, the plate was scored for live and dead worms. *C. elegans* with the PCMF was maintained to assess the toxicity, if any.

### Total phenolic content of PCMF

The total phenolic content of the PCMF was determined by the method of Spanos and Wrolstad (1990), as modified by Lister and Wilson (2001).

### Gas chromatography–mass spectrometry (GC–MS) analysis of PCMF

The compositions of the PCMF were analyzed by using the Perkin Elmer GC AutoSystem XL and TurboMass software as described previously by Husain et al. (2015a). The components were identified by the method described by Masada (1976). Quantitative data were obtained by the peak normalization technique using the integrated flame ionization detector (FID) response.

### Molecular docking analysis

The knowledge of protein three-dimensional (3D) structures are vital for rational drug design (Stephens et al., 2014; Khan et al., 2017a; Lan et al., 2017; Zhao et al., 2017). The 3D structure of RhlR was predicted using homology. Molecular docking studies were carried out to understand the proper positioning of drugs into the active pocket of a receptor to understand the mechanism of substrate binding and selectivity (Khan et al., 2015, 2016a). The molecular docking of bakuchiol was performed using LasR (PDB: 2UV0) and the homology-modeled structure of Rh1R as receptors. The 3D structure of bakuchiol was obtained from PubChem with compound identifier 5468522. The docking studies were performed to understand the bound confirmations and the binding affinity of bakuchiol with LasR and Rh1R. Bakuchiol was docked by describing the grid box with a spacing of 1 Å and size of 20 × 20 × 20, pointing in x, y, and z directions around the active pocket of protein following the standard docking protocol (Cosconati et al., 2010; Khan et al., 2017b) by using AutoDockTools and AutoDockVina (Trott and Olson, 2010) with default docking parameters. The Lamarckian genetic algorithm was selected as the search algorithm. The most apposite docked conformation was selected for the analysis. PyMol (Rigsby and Parker, 2016), Discovery Studio Visualizer (Biovia, 2015), and LigPlot^+^ (Laskowski and Swindells, 2011) were used for visualizing the docked complex. Further, the selected docked complex was subjected to molecular dynamics (MD) simulations to validate the stability of the docked complex.

### MD simulations

MD simulations were performed on the LasR, LasR–bakuchiol, Rh1R, and Rh1R–bakuchiol complexes using the GROMOS96 43a1 force-field at 300 *K* using GROMACS 5.1.2 (Van Der Spoel et al., 2005). Bakuchiol was extracted from the docked complexes such as LasR–bakuchiol and Rh1R–bakuchiol using the *gmx grep* command. The force-field parameter and the topology files of bakuchiol were generated using the PRODRG server (Schüttelkopf and van Aalten, 2004). The charges in the topology file were properly corrected. The topologies of LasR and Rh1R using the *pdb2gmx* modules of GROMACS, and that of bakuchiol using the PRODRG server were combined and a further 24 atoms of bakuchiol were included. The bakuchiol parameter was incorporated in the system topology file. The individual protein atoms and complexes were soaked with water molecules in a cubic box having a dimension of 10 Å, i.e., box edge of 10 Å from the molecule periphery. The modules *gmx editconf* and *gmx solvate* modules were used for creating the boundary conditions and for solvation, respectively. The simple point-charge (spc216) water model was used to solvate the protein and the complex.

The *gmx genion* module was used to counterbalance the charges on LasR and LasR–bakuchiol. The Rh1R and Rh1R–bakuchiol complexes were counterbalanced by the addition of Na^+^ and Cl^−^ ions to maintain neutrality and preserve a physiological concentration of 0.15 M. For the LasR–bakuchiol and Rh1R–bakuchiol complexes, bakuchiol was added to the energy groups of the molecular dynamics parameters (mdp) file, to inspect the interactions of bakuchiol with LasR and Rh1R, respectively. The final system was minimized using the steepest descent method, and the temperature was then elevated from 0 to 300 K during the equilibration period of 100 ps at a constant volume under periodic boundary conditions.

The restraints to the bakuchiol were applied during the NVT equilibration period using the *genrestr* module, and then the treatment of the temperature coupling groups. Two-phase equilibrations were achieved: the NVT ensemble with a constant number of particles, volume, and temperature at 100 ps, and the NPT ensemble with a constant number of particles, pressure, and temperature at 100 ps. The C^α^ backbone atoms of the structure were restrained, and all other atoms were allowed to move freely during equilibration steps. The particle-mesh Ewald method (Norberto de Souza and Ornstein, 1999) was applied after the equilibration steps, and the 100 ns production phases were carried out at 300 *K*. The results were analyzed using the *gmx energy, gmx rms, gmx confirms, gmx rmsf* , *gmx gyrate, make_ndx, gmx hbond, gmx do_dssp*, and *gmx sasa* utilities of GROMACS. The graphical presentations of the 3D models were prepared using Discovery Studio and Visual Molecular Dynamics (VMD) (Humphrey et al., 1996).

### Statistical analysis

All studies were performed in triplicate and the data obtained from experiments were presented as mean values and the differences between the control and the test were analyzed using a Student's *t*-test.

## Results and discussion

### Fraction-based screening for violacein inhibition in *C. violaceum*

Different fractions of *P. corylifolia* (seed) obtained in petroleum ether, benzene, ethyl acetate, acetone, and methanol were tested for their QS modulatory activity at varying concentrations against the *C. violaceum* ATCC 12472 (CV12472) strain. Fraction-based anti-QS activity against *C. violaceum* ATCC 12472 was demonstrated by the *P. corylifolia* methanol extract at 400 and 800 μg/ml concentrations, while at 1,600 μg/ml, pigment inhibition was accompanied by the inhibition of growth. Similarly, acetone and ethyl acetate extracts also demonstrated comparatively less pigment inhibition accompanied by growth inhibition. However, no activity was detected in petroleum ether and benzene fraction at all tested concentrations (Table 1).

**Table 1:** Pigment inhibitory activity of different fractions of *Psoralea corylifolia* (seed) extract.

**Name of the fraction**	**Concentration of extract (μg/ml)**	**Zone of inhibition against** ***C. violaceum*** **ATCC 12472 (CV12472) in mm**		
		**Total inhibition (r_1_)**	**Growth inhibition (r_2_)**	**Pigment inhibition (r_1_-r_2_)**
Petroleum ether	200	-	-	-
	400	-	-	-
	800	-	-	-
	1,600	-	-	-
Benzene	100	-	-	-
	200	-	-	-
	400	-	-	-
	800	-	-	-
Ethyl acetate	150	-	-	-
	300	-	-	-
	600	13	13	-
	1,200	13	9	4
Acetone	100	-	-	-
	200	19	17	2
	400	25	21	4
	800	27	25	2
Methanol	200	-	-	-
	400	15	-	15
	800	16	-	16
	1,600	18	3	15

*Data are the mean value of three experiments*.

- Shows no activity

*Total inhibition = total zone of pigment inhibition including growth inhibition, if any*.

The MIC of the *P. corylifolia* methanol fraction was determined against all test pathogens. An MIC of 750 μg/ml was observed against *C. violaceum* CVO26, *S. marcescens*, and *L. monocytogenes*, while a concentration of 1,250 μg/ml was recorded for *P. aeruginosa* PAF79 and *A. hydrophila* WAF38. The highest MIC of 1,500 μg/ml was observed against PAO1. Concentrations below the MICs i.e., sub-MICs were considered for all assays on the QS-regulated virulence functions and the biofilm.

**Figure 1:**
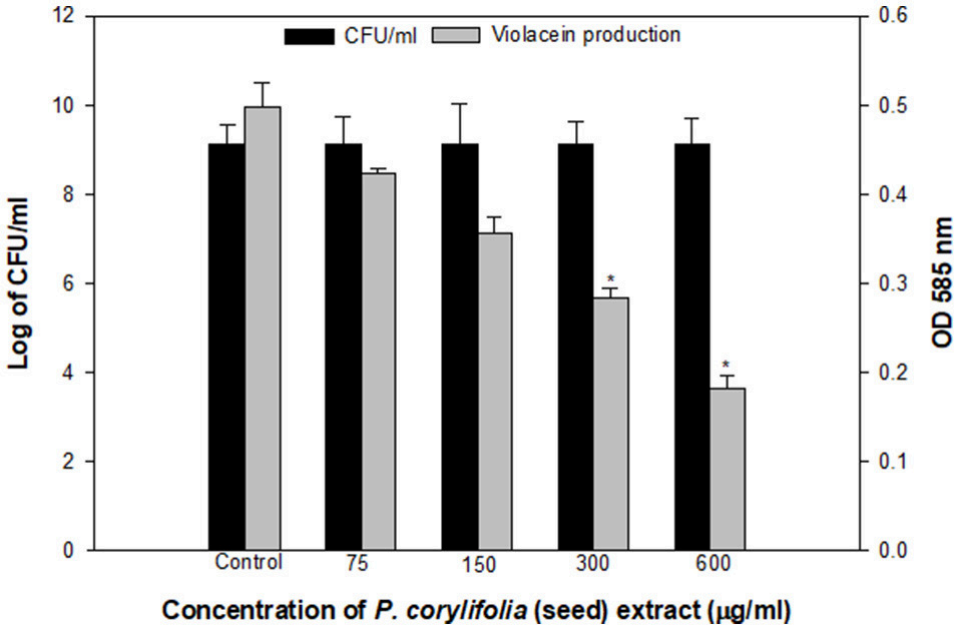
Quantitative assessment of violacein inhibition in CVO26 by sub-MICs of *P. corylifolia* (seed) extract. All the data are presented as mean ± SD. * significance at *p* ≤ 0.05.

The QS inhibitory activity of the methanol fraction of *P. corylifolia* (seed) was confirmed by determining the extent of violacein production in *C. violaceum* CV026, a mutant strain of wild-type CV12472 as depicted in Figure 1. The extract exhibited a significant reduction in violacein production and this reduction increased with the increasing concentration of the PCMF. A maximum reduction of 63.3% over control was observed at a concentration of 600 μg/ml of the extract. An insignificant difference in the number of colony-forming units (CFU) was recorded. Violacein production in *C. violaceum* is regulated by the CviIR-dependent QS system. Therefore, any inhibition of the pigment in CVO26 is indicative of the fact that the extract is acting on the CviIR QS system and is a direct evidence of QS interference. Similar dose-dependent inhibition of violacein in CVO26 has been demonstrated in the extracts of *Terminalia chebula* (Sarabhai et al., 2013), *T. foenum-graceum* (Husain et al., 2015a), *Centella asiatica* (Vasavi et al., 2016), and *M. indica* (Husain et al., 2017).

### Effect on QS-regulated functions in *P. aeruginosa*

QS interference by the methanol extract of *P. corylifolia* (seed) against *P. aeruginosa* strains is presented in Tables 2, 3. The data showed a statistically significant reduction in the LasB elastolytic activity of PAO1 and PAF79 by 49.7 and 46.1%, respectively. Similarly, the total proteolytic activity was reduced by 50.5% in PAF79 and 43.5% in PAO1 at the respective sub-MICs. Proteases and LasB play a major role in the pathogenesis of *P. aeruginosa* by degrading the host tissues (Kessler et al., 1993). The virulence factor LasB (elastase) is controlled both by the *lasI*–*lasR* and *rhlI*–*rhlR* systems (Brint and Ohman, 1995; Pearson et al., 1997; Hentzer and Givskov, 2003). Our findings are in agreement with previous reports on the extracts of *Ananas comosus, Musa paradiciaca, Manilkara zapota, Ocimum santum, Lagerstroemia speciosa*, and *Allium cepa* (Musthafa et al., 2010; Singh et al., 2012; Vasavi et al., 2016; Al-Yousef et al., 2017).

**Table 2:** Effect of sub-MICs of methanolic extract of *Psoralea corylifolia* (seed) on inhibition of quorum sensing-regulated virulence factors in *P. aeruginosa* PAO1.

**Extract concentration (μg/ml)**	**Elastase activity^a^**	**Total protease^b^**	**Pyocyanin production^c^**	**Chitinase activity^d^**	**EPS production^e^**	**Swarming motility^f^**
Control	0.181 ± 0.044	1.420 ± 0.038	5.2 ± 0.6	0.120 ± 0.009	0.991 ± 0.045	72 ± 1.5
125	0.156 ± 0.021 (13.8)	1.075 ± 0.036 (24.2)	2.45 ± 0.19 (52.8)^*^	0.082 ± 0.011 (31.6)	0.754 ± 0.049 (23.9)	46 ± 1.45 (36.1)
250	0.115 ± 0.013 (36.4)	1.01 ± 0.025 (28.8)	1.72 ± 0.33 (66.9)^**^	0.048 ± 0.017 (60)^**^	0.700 ± 0.018 (29.3)	37 ± 2.0 (48.6)^*^
500	0.101 ± 0.006 (44.1)	0.938 ± 0.019 (33.9)	1.5 ± 0.22 (71.1)^**^	0.040 ± 0.005 (66.6)^**^	0.515 ± 0.027 (48.0)^*^	29 ± 0.80 (59.7)^*^
1,000	0.091 ± 0.009 (49.7)^*^	0.801 ± 0.007 (43.5)^*^	0.69 ± 0.10 (86.7)^***^	0.029 ± 0.005 (75.8)^***^	0.429 ± 0.025 (56.7)^*^	26 ± 1.2 (63.8)^*^

a *Elastase activity is expressed as the absorbance at OD_495_*.

b *Total protease activity is expressed as the absorbance at OD_600_*.

c *Pyocyanin concentrations were expressed as micrograms of pyocyanin produced per microgram of total protein*.

d *Chitinase activity is expressed as the absorbance at OD_570_*.

e *EPS production is expressed as absorbance at OD_480_*.

f *Swarming motility is expressed as diameter of swarm in mm*.

All the data are presented as mean ± SD.

* *significance at p ≤ 0.05*,

*Values in the parentheses indicate percent reduction over control*.

**Table 3:** Effect of sub-MICs of methanolic extract of *Psoralea corylifolia* (seed) on inhibition of quorum sensing-regulated virulence factors in *P. aeruginosa* PAF-79.

**Extract concentration (μg/ml)**	**Elastase activity^a^**	**Total protease^b^**	**Pyocyanin production^c^**	**Chitinase activity^d^**	**EPS production^e^**	**Swarming motility^f^**
Control	0.167 ± 0.025	1.039 ± 0.041	3.8 ± 0.25	0.139 ± 0.005	0.886 ± 0.036	48 ± 1.5
100	0.148 ± 0.004 (11.3)	0.938 ± 0.021 (9.7)	3 ± 0.2 (21)	0.114 ± 0.008 (17.9)	0.661 ± 0.015 (25.3)	40 ± 0.5 (16.6)
200	0.140 ± 0.015 (16.1)	0.891 ± 0.030 (14.2)	2.4 ± 0.13 (36.8)	0.07 ± 0.005 (49.6)*	0.525 ± 0.018 (40.6)	33 ± 2 (31.2)*
400	0.115 ± 0.007 (31.1)	0.748 ± 0.014 (28)	2.1 ± 0.082 (44.7)*	0.063 ± 0.007 (54.6)*	0.373 ± 0.012 (57.9)*	24 ± 1.5 (50)*
800	0.090 ± 0.003 (46.1)*	0.515 ± 0.012 (50.5)*	1.6 ± 0.054 (57.8)*	0.051 ± 0.008 (63.3)*	0.292 ± 0.014 (67)*	22 ± 2.5 (54.1)*

a *Elastase activity is expressed as the absorbance at OD_495_*.

b Total protease activity is expressed as the absorbance at OD_600._

c Pyocyanin concentrations were expressed as micrograms of pyocyanin produced per microgram of total protein.

d Chitinase activity is expressed as the absorbance at OD_570_.

e EPS production is expressed as absorbance at OD_480_.

f Swarming motility is expressed as diameter of swarm in mm.

*All the data are presented as mean ± SD*.

* significance at p ≤ 0.05.

*Values in the parentheses indicate percent reduction over control*.

Pyocyanin production is regulated by QS and causes severe toxic effects in humans by inducing the apoptosis of neutrophils and damaging the neutrophil-mediated host defense (Fothergill et al., 2007). Pyocyanin production was reduced significantly at all concentrations in PAO1. However, in PAF79, pyocyanin production was reduced maximally to 57.8% over untreated control at a concentration of 800 μg/ml. The inhibition of pyocyanin by sub-MICs of the PCMF is an important finding, considering the role of pyocyanin in the pathogenesis of *P. aeruginosa*. Similar concentration-dependent results were observed with the *T. foenum-graceum* seed extract, leaf extracts of *Piper betle* and *M. indica*, and *Forsythia suspensa* extract (Husain et al., 2015a, 2017; Datta et al., 2016; Zhang and Chu, 2017).

Chitinase activity in both the strains of *P. aeruginosa* was impaired significantly upon treatment with sub-MICs of the PCMF. In PAO1, 31.6–75.8% reduction in chitinase was observed while in PAF79, the decrease in chitinase production ranged from 17.9 to 63.3% over untreated control (Tables 2, 3). This significant reduction in chitinase produced by the *P. aeruginosa* strains after treatment with sub-MICs of the PCMF corroborates well with the findings on *T. foenum-graceum* (21–48% reduction) and *M. indica* (21–55%) (Husain et al., 2015a, 2017).

**Figure 2:**
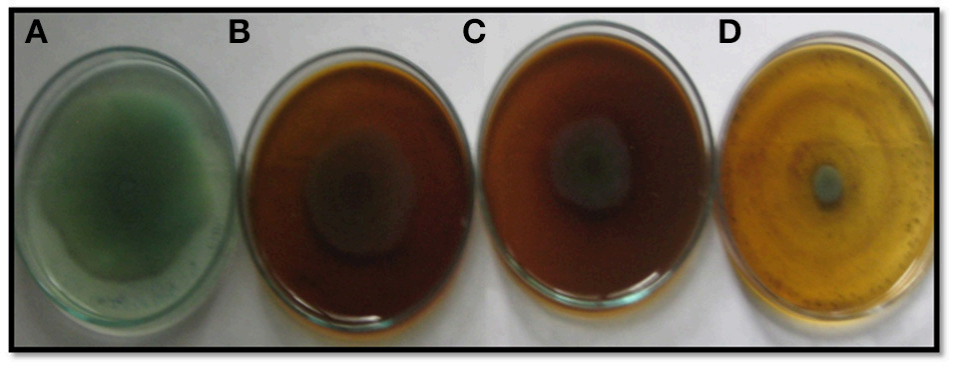
Inhibition of swarming motility in *P. aeruginosa* PAF79 by sub-MICs of methanol extract of *P. corylifolia* (seed), **(A)** Untreated control; **(B)** 200 μg/ml; **(C)** 400 μg/ml; **(D)** 800 μg/ml.

EPS and swarming motility are vital at various stages of biofilm formation. EPS protects the biofilm from antimicrobial agents and is important during the maturation of the biofilm. Motility is essential during the initial attachment of the cells to the surface (Rabin et al., 2015). Sub-MICs of the PCMF effectively interfered with the production of EPS in PAO1 and PAF79. Swarming motility was also reduced substantially in both the test strains at the respective sub-MICs as depicted in Tables 2, 3 and Figure 2. Since EPS and swarming motility are crucial to biofilm formation, it is envisaged that the PCMF at sub-inhibitory concentrations will decrease the biofilm-forming capabilities of the test pathogens.

### Effect on QS-regulated functions in *A. hydrophila*

The extract of *P. corylifolia* (100–800 μg/ml) effectively interfered with the QS-regulated traits of *A. hydrophila* WAF38 and showed a significant reduction in the total protease activity to the level of 39.5–65.5% (*p* ≤ 0.005) without affecting the growth significantly (Figure S1A). Similar concentration-dependent decrease (29.1–69.9%) in EPS production was also recorded at the tested sub-MICs of the PCMF (Table 4). The production of EPS and proteases in *A. hydrophila* is regulated by the *ahyRI* QS system. The decrease in the production of total proteases and EPS indicates that the PCMF interferes with the *ahyRI* QS system of *A. hydrophila* and consequently impairs C4-HSL production.

**Table 4:** Effect of sub-MICs of methanolic extract of *Psoralea corylifolia* (seed) on inhibition of quorum sensing-regulated virulence factors in *Aeromonas hydrophila* WAF-38.

**Concentration (μg/ml)**	**Total protease^a^**	**EPS production^b^**
Control	0.589 ± 0.051	0.748 ± 0.021
100	0.356 ± 0.016 (39.5)	0.530 ± 0.039 (29.1)
200	0.298 ± 0.029 (49.4)*	0.364 ± 0.026(51.3)*
400	0.278 ± 0.010 (52.8)*	0.29 ± 0.013 (61.2)**
800	0.203 ± 0.004 (65.5)**	0.255 ± 0.013 (69.9)**

a *Total protease activity is expressed as the absorbance at OD_600_*.

b EPS production is expressed as absorbance at OD_480_.

*All the data are presented as mean ± SD*.

* *significance at p ≤ 0.05*,

** , significance at p ≤ 0.005.

*Values in the parentheses indicate percent reduction over control*.

### Effect on prodigiosin production in *serratia marcescens*

**Figure 3:**
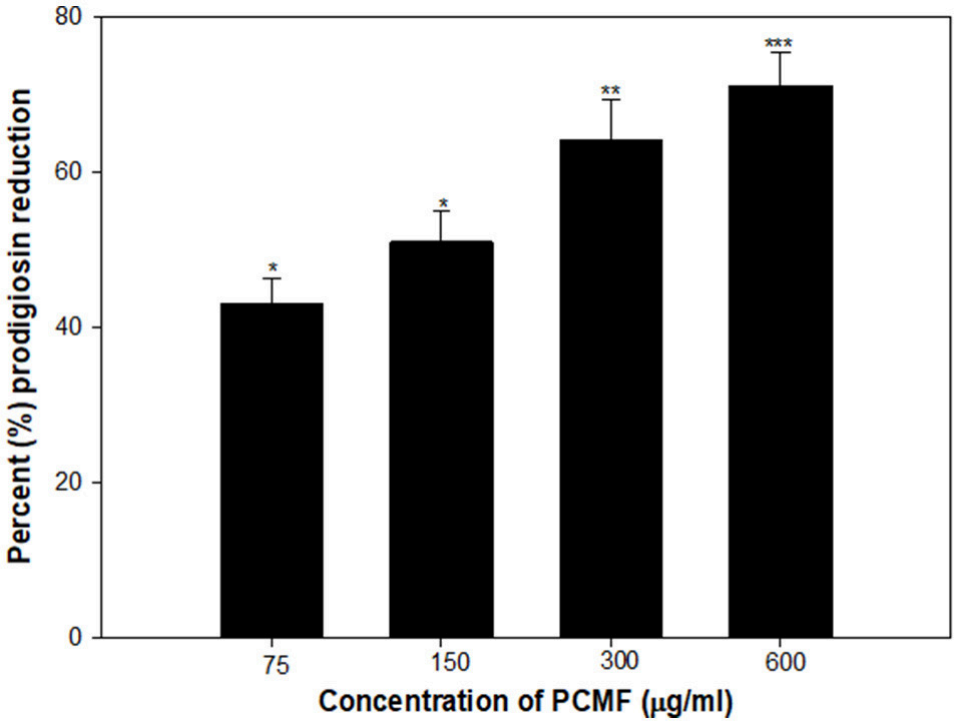
Quantitative assessment of Prodigiosin inhibition in *S. marcescens* by sub-MICs of PCMF. All the data are presented as mean ± SD. * significance at *p* ≤ 0.05, ** significance at *p* ≤ 0.005, *** significance at *p* ≤ 0.001.

A dose-dependent decrease in the production of prodigiosin by *S. marcescens* was recorded at the sub-MICs ranging from 75 to 600 μg/ml. The reduction was statistically significant (*p* ≤ 0.005) at all the sub-inhibitory concentrations tested (Figure 3). The maximum inhibition of 71% and the lowest of 43% were recorded at concentrations of 600 and 75 μg/ml of the PCMF, respectively. The growth of the pathogen was not inhibited significantly (Figure S1B). Prodigiosin is considered as a major virulence factor of *S. marcescens* and is QS-regulated (Morohoshi et al., 2007). Hence, it is envisaged that the inhibition of prodigiosin will reduce the pathogenicity of *S. marcescens*. Methanol extracts of *Anethum graveolens* and three marine sponges have been previously reported for similar concentration-dependent reduction of prodigiosin (Annapoorani et al., 2012; Salini and Pandian, 2015).

### Effect on pcmf on biofilm formation

**Figure 4:**
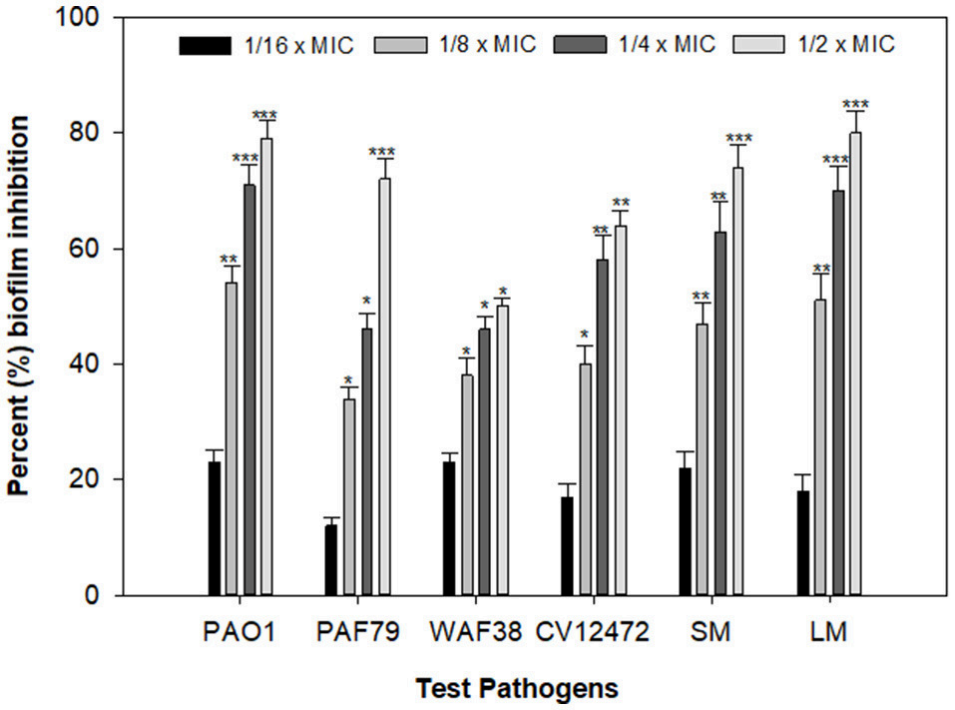
Effect of PCMF on biofilm formation of test bacterial pathogens as quantified by crystal violet staining. Data are represented as the percentage inhibition of biofilm formation. All the data are presented as mean ± SD. * significance at *p* ≤ 0.05, ** significance at *p* ≤ 0.005, *** significance at *p* ≤ 0.001.

Biofilms are cells growing in a self-produced matrix of EPS, which protects the encapsulated bacteria from the external environment and increases their resistance against antimicrobial agents many folds (Aitken et al., 2011). Reports have suggested that the negative charge on the polymers of the biofilm matrix interacts with positively charged antibiotics such as the aminoglycoside group of antibiotics and hampers the entry of such antibacterial drugs (Stewart and Costerton, 2001). In the present study, the PCMF significantly reduced biofilm formation in all the selected human- and food-related pathogens at the respective sub-MICs. Maximum reductions of 79, 71, 50, 64, 77, and 80% in the biofilm-forming capability of *P. aeruginosa* PAO1, *P. aeruginosa* PAF79, *A. hydrophila* WAF38, *C. violaceum* 12472, *S. marcescens*, and *L. monocytogenes* were observed over untreated control, respectively (Figure 4). Similar observations have been recorded with *Capparis spinosa* (Issac Abraham et al., 2011), *Rosa rugosa* (Zhang et al., 2014), leaf extract of *Kalanchoe blossfeldina* (Sarkar et al., 2015), and onion peel extract (Al-Yousef et al., 2017), which are known to reduce biofilm formation in pathogenic bacteria.

### Effect of on β-galactosidase activity

**Figure 5:**
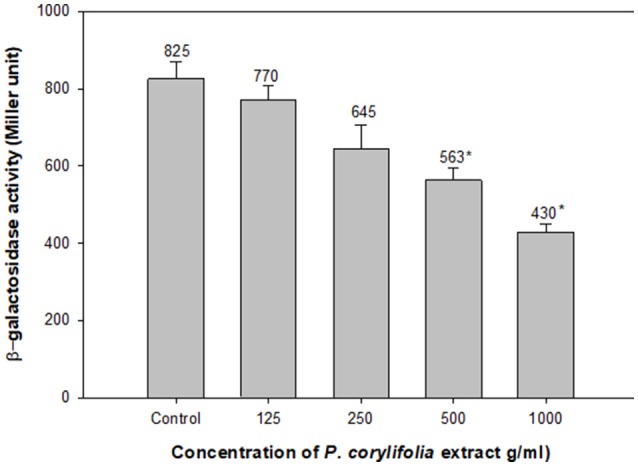
Effect of PCMF on β-galactosidase activity in *E. coli* MG4/pKDT17. All the data are presented as mean ± SD. * significance at *p* ≤ 0.05.

The effect of the *P. corylifolia* (seed) extract (125–1,000 μg/ml) was also assessed on the levels of the AHL produced by PAO1 using the β-galactosidase activity of *E. coli* MG4/pKDT17. A dose-dependent decrease was recorded for all the sub-MICs tested and a significant reduction of 47.8% was observed at 1,000 μg/ml as shown in Figure 5. The results of the β-galactosidase assay suggest that the quorum-sensing and biofilm-inhibitory activities of the PCMF were initiated by the downregulation of *las*-controlled transcription by sublethal concentrations of the PCMF.

### Assessment of anti-infective potential of PCMF in *C. elegans* nematode model

**Figure 6:**
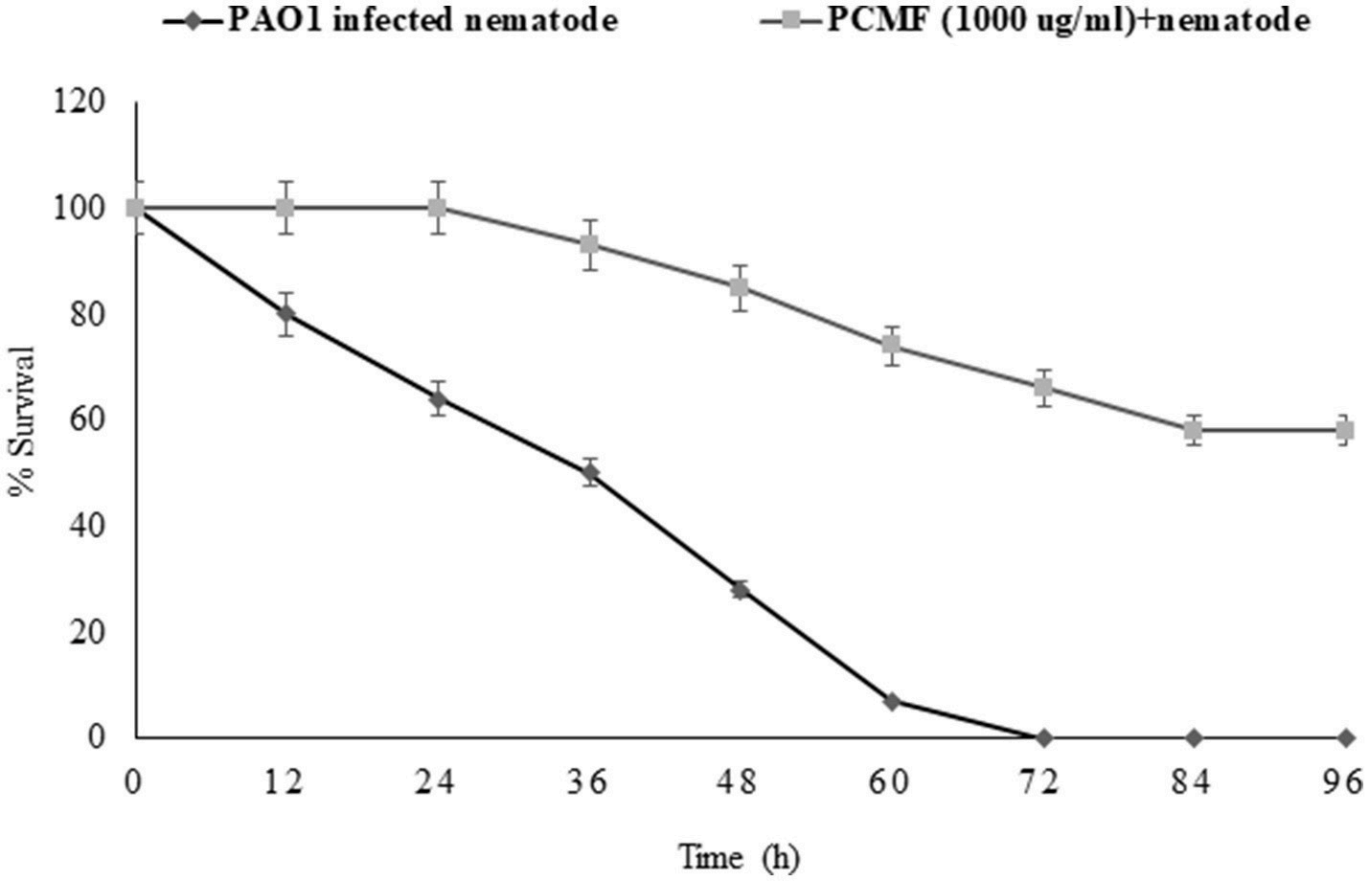
Anti-infection potential of sub-MIC (1,000 μg/ml) of methanol fraction of *P. corylifolia* (seed) against PAO1-preinfected *C. elegans* nematode model. Means values and SDs are shown.

The findings of the *in vitro* assays were also investigated *in vivo* using the liquid killing assay in the *C. elegans* nematode model. Potent pathogenicity of PAO1 toward the *C. elegans* nematode was observed as all the preinfected nematodes died within 72 h of the infection. However, preinfected *C. elegans* treated with *P. corylifolia* (1,000 μg/ml) displayed an enhanced survival rate of 58% (Figure 6). Methanol alone did not cause any significant mortality of the nematodes. *P. aeruginosa* PAO1 kills the nematodes by causing cyanide asphyxiation and paralysis (Gallagher and Manoil, 2001). The increased survival of preinfected nematodes treated with 1,000 μg/ml of the PCMF suggests that the extract interferes with the QS system of PAO1, leading to reduction in deaths of the nematodes. The outcome of the *in vivo* studies are in accordance with the reports on South Florida plants, *Murraya koengii* essential oil, and *M. indica* (Adonizio et al., 2008b; Ganesh and Rai, 2016; Husain et al., 2017).

### Total phenolic content

The total phenolic content of various fractions (mg/g of dry extract) was determined as the gallic acid equivalent (GAE) by the Folin–Ciocalteu method. The methanol fraction of seed contained 367.6 ± 1.5 mg GAE/g of dry extracts followed by acetone (337.6 ± 1.4), ethyl acetate (292 ± 2.3), benzene (43.3 ± 1.1), and petroleum ether (43.1 ± 1.0) fractions.

### GC–MS analysis

A total of 21 chemical components were identified in the seed extract by GC–MS analysis. These numbers may be extended with the help of chemometric techniques. The major compounds identified were 9,12-Octadecadienoic acid (35.72%), followed by bakuchiol (27.73%), palmitic acid (23.12%), and myristic acid (1.050%). The percentages of the remaining compounds ranged from 0.1 to 0.5 as presented in Table 5.

**Table 5:** Components of *Psoralea corylifolia* (seed) extract as identified by GC–MS analysis.

**Peak no**.	**Components**	**Retention time**	**Area (%)**
1.	Trans(β)-caryophyllene	8.58	0.22
2.	1-Heptatriacotanol	10.69	0.29
3.	Caryophyllene oxide	11.32	0.48
4.	3-Methyl-5-(2,6,6-trimethyl-1-cyclohexen-1-yl)-1-pentyn-3-ol	11.60	0.64
5.	3-Ethyl-3-hydroxyandrostan-17-one	11.75	0.25
6.	Myristic acid	12.64	1.05
7.	3,7,11,15-Tetramethyl-2-hexadecen-1-ol	13.40	0.35
8.	Palmitic acid, methyl ester	14.29	0.57
9.	Palmitic acid	14.87	23.12
10.	Bakuchiol	16.37	27.73
11.	9,12-Octadecadienoic acid	16.66	35.72
12.	Linalol oxide, trimethylsilyl ether	21.67	0.20
13.	Squalene	24.80	0.11
14.	Hexacosane	25.71	0.20
15.	(+)-cis-Longipinane	25.84	0.42
16.	γ-Tocopherol	27.34	0.34
17.	Thunbergol	27.62	0.37
18.	Cholesteryl myristate	27.87	0.24
19.	Stigmasterol	29.00	0.57
20.	γ –Sitosterol	29.49	0.19
21.	trans-Longipinocarveol	29.60	0.22

### Evaluation of quorum sensing inhibitory activity of bakuchiol

**Figure 7:**
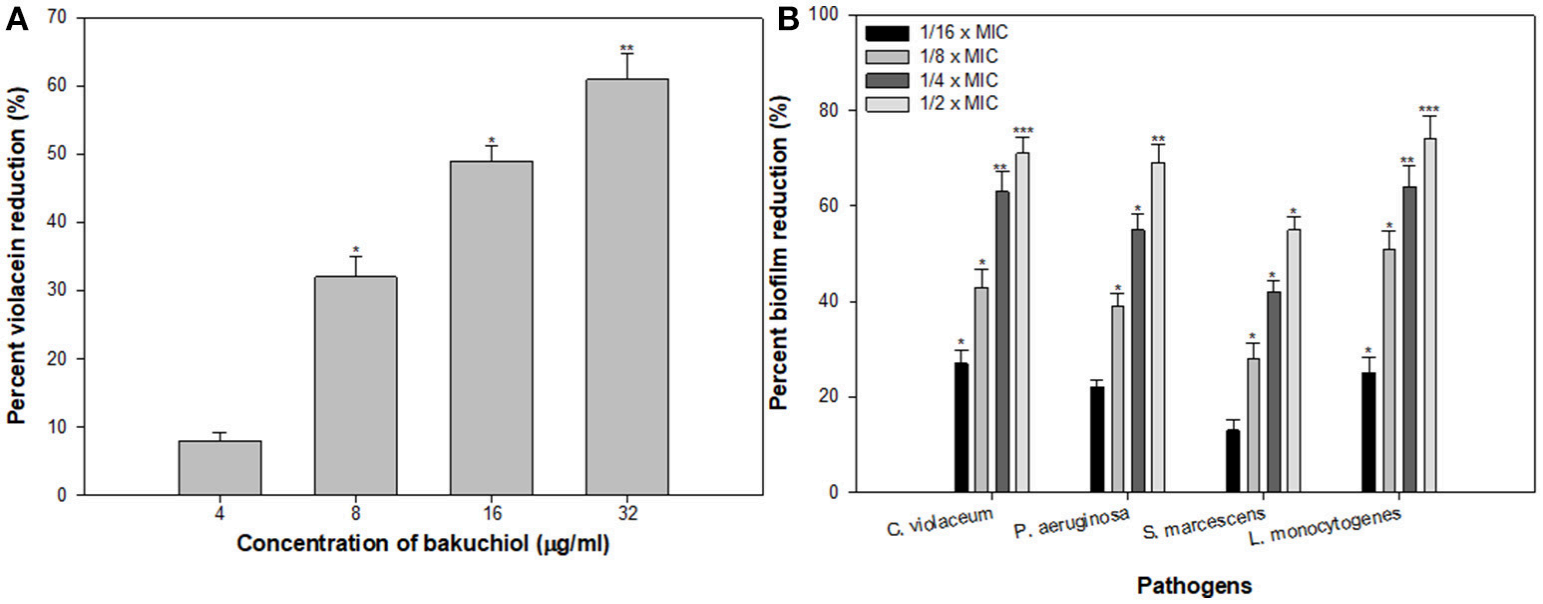
**(A)** Inhibition of violacein in *C. violaceum* by sub-MICs of bakuchiol. **(B)** Effect of bakuchiol on biofilm formation by the test pathogens. All the data are presented as mean ± SD. * significance at *p* ≤ 0.05, ** significance at *p* ≤ 0.005, *** significance at *p* ≤ 0.001.

**Figure 8:**
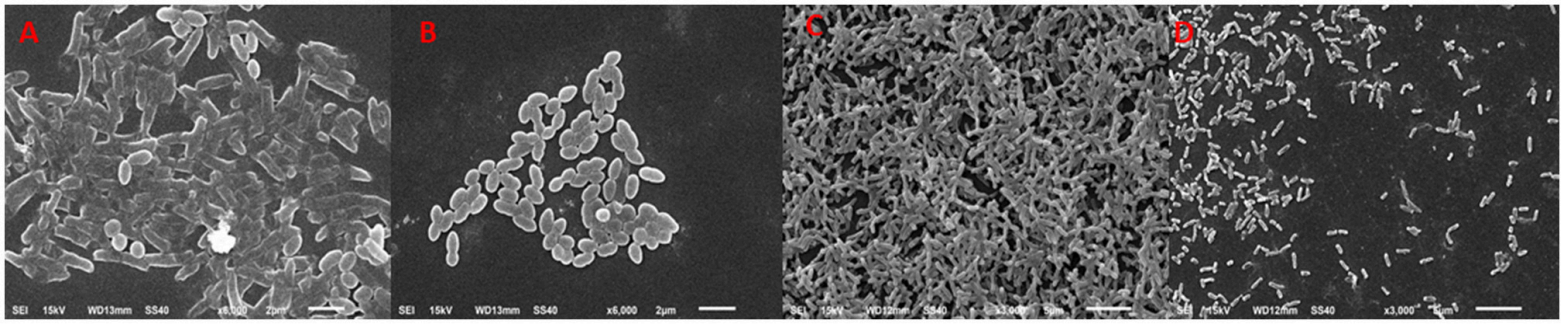
Scanning electron microscopic images demonstrating biofilm inhibition by sub-MICs of bakuchiol. **(A,C)** untreated control of *L. monocytogenes* and *P. aeruginosa*, respectively; **(B,D)** inhibition of biofilm formed by *L. monocytogenes* and *P. aeruginosa*, respectively by $\frac{1}{2}$ × MIC of bakuchiol.

Since bakuchiol was found to be the chief phytoconstituent present in the PCMF, it was assessed for anti-QS and anti-biofilm potential *in vitro* using *C. violaceum* CVO26, *P. aeruginosa* PAO1, *S. marcescens*, and *L. monocytogenes*. The MIC of bakuchiol was found to be 64, 128, 32, and 64 μg/ml against *C. violaceum* CVO26, *P. aeruginosa* PAO1, *S. marcescens*, and *L. monocytogenes*, respectively. At the tested sub-MICs (4–32 μg/ml), bakuchiol demonstrated statistically significant inhibition of the violacein pigment ranging from 8 to 61% over untreated control (Figure 7A). The biofilm formation by PAO1 was also impaired by 22, 39, 55, and 69% at 8, 16, 32, and 64 μg/ml concentrations, respectively (Figure 7B). Further, bakuchiol significantly reduced the biofilm-forming capabilities of *C. violaceum* CV12472, *S. marcescens*, and *L. monocytogenes* at the respective sub-MICs. Biofilm formation in *C. violaceum* ATCC 12472 was reduced by 27–71% at concentrations ranging from 4 to 32 μg/ml (Figure 7B), while the biofilm formed by *S. marcescens* and *L. monocytogenes* decreased by 13–55% and 25–74%, respectively (Figure 7B). Scanning electron microscopic images demonstrated significant reduction in the number of microcolonies of *P. aeruginosa* and *L. monocytogenes* after treatment with $\frac{1}{2}$× MIC of bakuchiol (Figures 8A–D). In a similar study, quercetin 4′-O-β-D glucopyranoside, without impacting the growth of pathogens such as *C. violaceum* 12472, *P. aeruginosa* PAO1, *S. marcescens*, and *L. monocytogenes*, significantly inhibited (*P* < 0·05) the biofilm formation and production of virulence factors including pyocyanin, protease, and elastase at sublethal doses (Al-Yousef et al., 2017). Further, our findings are in accordance with other results published on methyl eugenol (Abraham et al., 2012), eugenol (Zhou et al., 2013), carvacrol (Burt et al., 2014), caffeine (Husain et al., 2015a), menthol (Husain et al., 2015b), and coumarins (D'Almeida et al., 2017). Owing to the previous report on QS inhibition by palmitic acid and linoleic acid (Widmer et al., 2007), it is envisaged that the QS inhibitory property of the PCMF is due to the presence of palmitic acid, linoleic acid, and bakuchiol.

### Molecular docking studies

**Figure 9:**
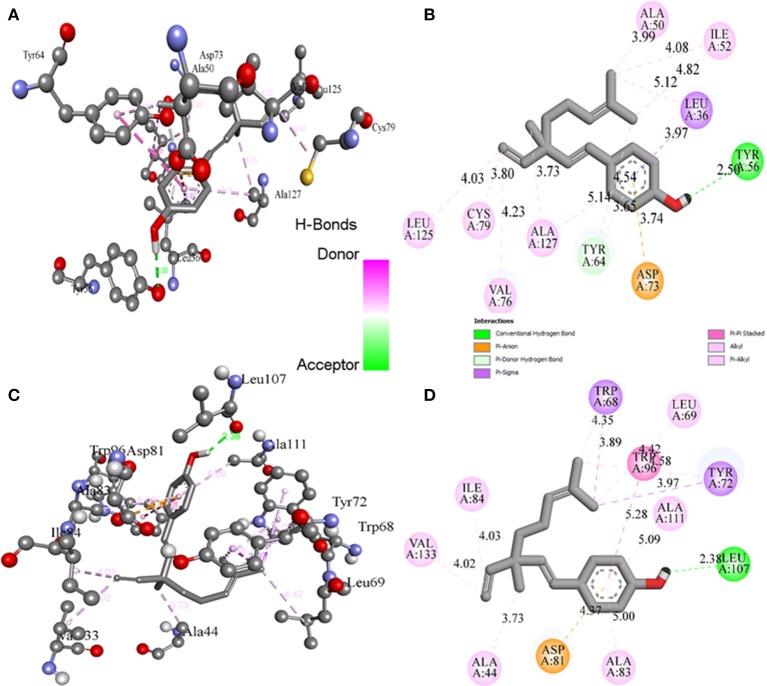
Binding of bakuchiol to the LasR and Rh1R estimated by molecular docking. **(A,B)** 3D and 2D representation of LasR showing the interaction with bakuchiol. **(C,D)** 3D and 2D representation of Rh1R showing the interaction with bakuchiol. Residues of LasR and Rh1R interact with bakuchiol were shown by ball and stick.

Molecular docking studies revealed the preferred positioning of bakuchiol in the active site of LasR and Rh1R. Bakuchiol binds in the active site cavity of LasR and Rh1R with a reasonable binding energy of −8.6 and −8.6 kcal/mol, respectively. The docked conformations indicate that bakuchiol binds into the cavity, and possibly inhibits LasR and Rh1R, and this may account for the modulation of its biological functions. The orientation of bakuchiol and a detailed interaction with the active site residues of LasR and Rh1R are shown in Figure 9. Bakuchiol was further examined on the basis of Lipinski's rule and the parameters calculated are listed in Table 6, demonstrating the drug-likeness of bakuchiol that can be implicated in LasR and Rh1R after further validation and optimization. The docked complexes were subjected to MD simulations to check the stability and the validity of the complexes. Four systems were prepared for each 100 ns MD simulation.

**Table 6:** Physiochemical properties of bakuchiol based on Lipinski's rule of 5 and showing drug likeliness*.

**Ligand**	**Molecular weight**	**iLOGP**	**H bond donor**	**H bond acceptor**	**Rotatable bond**	**Bioavailability score**	**Drug likeness**
Bakuchiol	256.38 (g/mol)	3.54	1	1	6	0.55	Yes

* *http://www.swissadme.ch/*.

### MD analysis

#### Potential energy

The MD simulation trajectories of LasR, LasR–bakuchiol, Rh1R, and Rh1R–bakuchiol were examined. To establish the equilibrium between systems tested earlier and MD data analysis, the average potential energy and the average fluctuation of temperature were checked. A constant continual temperature fluctuation at 300 *K* for each system was found to produce stable and accurate MD simulation results. The average potential energy for the LasR, LasR–bakuchiol, Rh1R, and Rh1R–bakuchiol complexes were found to be −586038.00, −585598.00, −1145220.00, and −1144500.00 kJ/mol, respectively.

#### Conformational changes in LasR and RhlR

**Figure 10:**
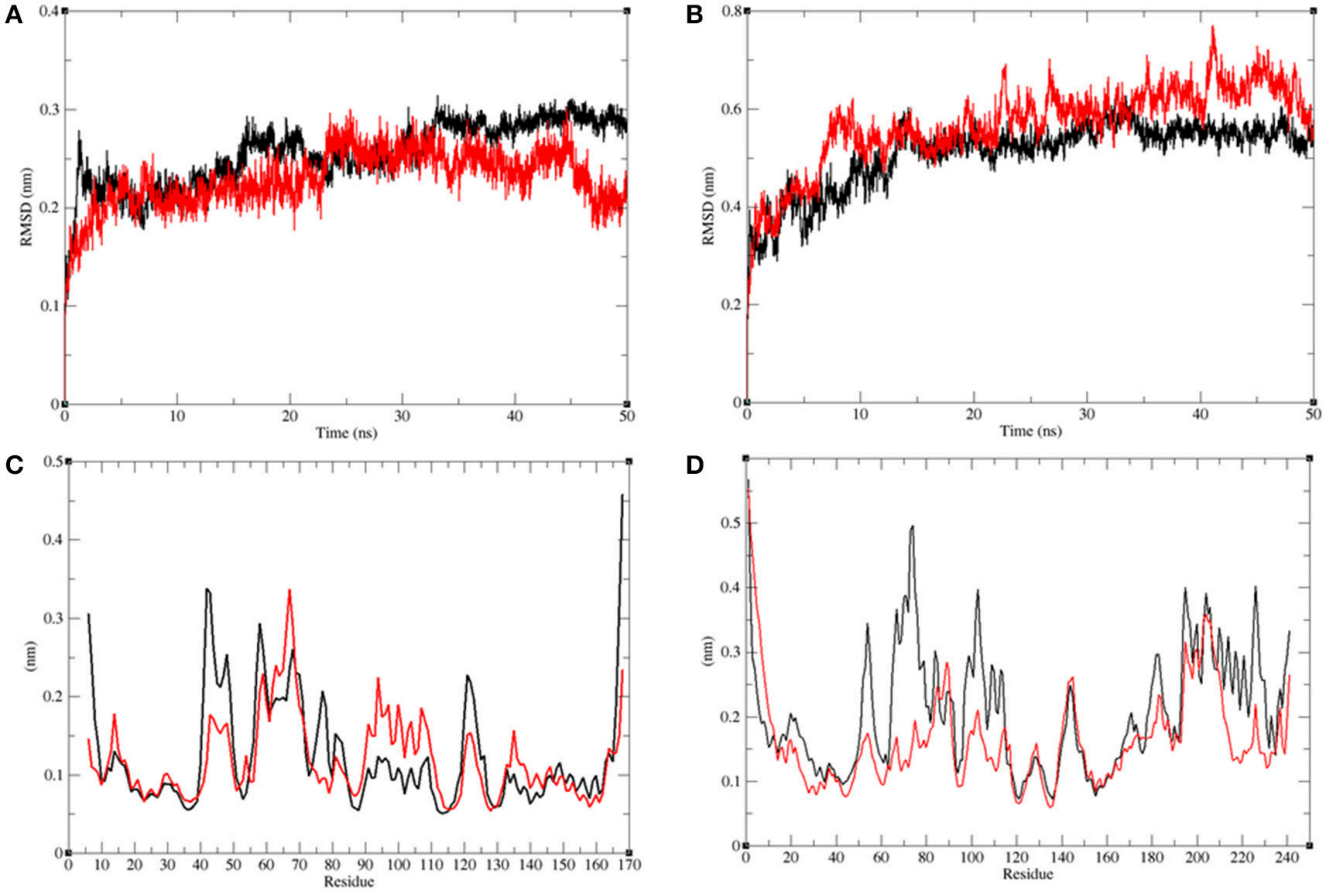
Analysis of conformational changes in the LasR and RhlR after binding of bakuchiol. **(A)** Plot of RMSD as a function of time obtained for unbound LasR (black), LasR**–**bakuchiol (red), respectively. **(B)** Plot of RMSD as a function of time obtained for unbound RhlR (black), RhlR**–**bakuchiol (red), respectively. **(C)** Plot of RMSF as a function of residues number obtained for unbound LasR (black), LasR**–**bakuchiol (red), respectively. **(D)** Plot of RMSF as a function of residues number obtained for unbound RhlR (black), RhlR**–**bakuchiol (red), respectively.

The structural comparison between protein molecules is an important tool for the analysis of protein structures and folding (Gramany et al., 2016; Khan et al., 2016b, 2018; Naz et al., 2018; Syed et al., 2018). The average root-mean-square deviation (RMSD) values were 0.20–0.30 nm for the LasR and LasR–bakuchiol complexes, respectively. The RMSD value of LasR decreased upon the binding of bakuchiol to the active pocket (Figure 10A). The RMSD trajectories suggested that LasR deviated from its native conformation upon binding to bakuchiol. Accordingly, the binding of bakuchiol to RhlR led to random fluctuations in the RMSD trajectories that arise due to structural deviations (Figure 10B).

The residual vibrations around the equilibrium are not accidental but governed by local structure flexibility. To determine the average fluctuation of all residues during the MD simulation, the root-mean-square fluctuation (RMSF) of the LasR, LasR–bakuchiol, Rh1R, and Rh1R–bakuchiol complexes were plotted as a function of residue number. The RMSF plot of LasR showed the least fluctuations at 40–60 amino acid (*aa*) residues; thereafter, it showed comparatively large fluctuations at 60–70 *aa* and 90–110 *aa* residues upon binding with bakuchiol. These fluctuations arose due to the binding of bakuchiol, thus leading to the structural deviations of LasR (Figure 10C). The binding of bakuchiol to RhlR minimized the residual fluctuations, and this may be attributed to the strong binding of bakuchiol to the active pocket of RhlR (Figure 10D).

#### Structural compactness

**Figure 11:**
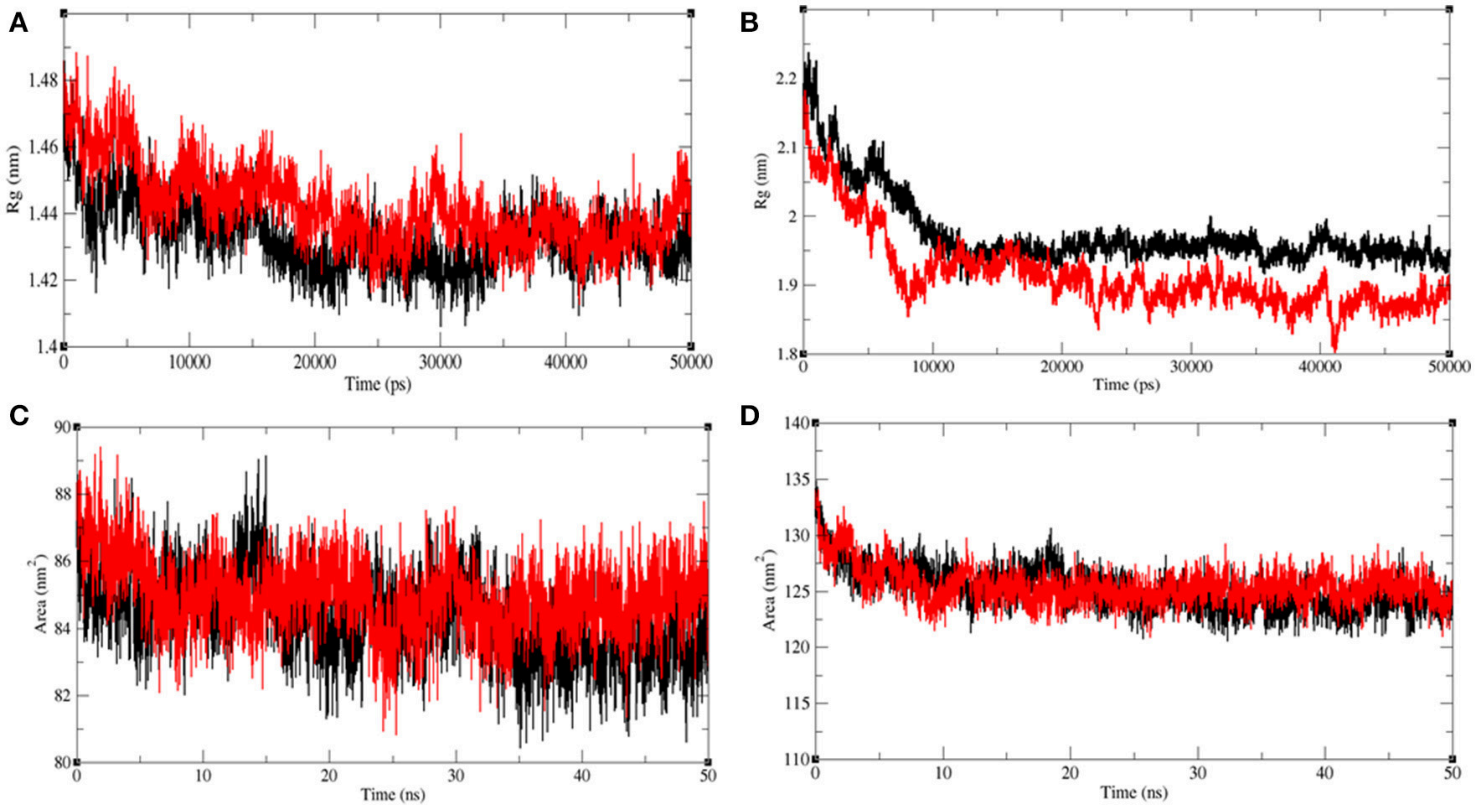
Analysis of compactness in the LasR and RhlR after binding of bakuchiol. Radius of gyration obtained for **(A)** unbound LasR (black), LasR**–**bakuchiol (red), and **(B)** unbound RhlR (black), RhlR**–**bakuchiol (red), respectively. Solvent accessible surface area as a function of time obtained **(A)** unbound LasR (black), LasR**–**bakuchiol (red), and **(B)** unbound RhlR (black), RhlR**–**bakuchiol (red), respectively.

The radius of gyration (R_*g*_) is related to the tertiary structure of a protein molecule. R*g* is calculated to determine the protein stability in a biological system. Higher values of Rg suggest loose packing in the protein structure and vice versa. The average R_*g*_ value for LasR was found to be higher upon bakuchiol binding (Figure 11A). We observed that the structure of LasR is relatively compact in the free state, but the binding of bakuchiol leads to slight deviations from its native conformations. Additionally, the average compactness of Rh1R changes slightly upon bakuchiol binding (Figure 11B).

Solvent-accessible surface area (SASA) is the surface area of a molecule that interacts with the solvent molecules (Mazola et al., 2015). The average SASA values for the LasR, LasR–bakuchiol, Rh1R, and Rh1R–bakuchiol complexes were calculated using the *gmx sasa* module of GROMACS. It was found that the average SASA values for LasR and RhlR when bound to bakuchiol were slightly higher than that in the unbound state. This is possibly due to the exposure of the internal residues in LasR and RhlR to the solvent due to the denaturation or conformational changes in the protein, arising due to the inhibition by bakuchiol (Figures 11C,D).

#### Secondary structure analysis

**Figure 12:**
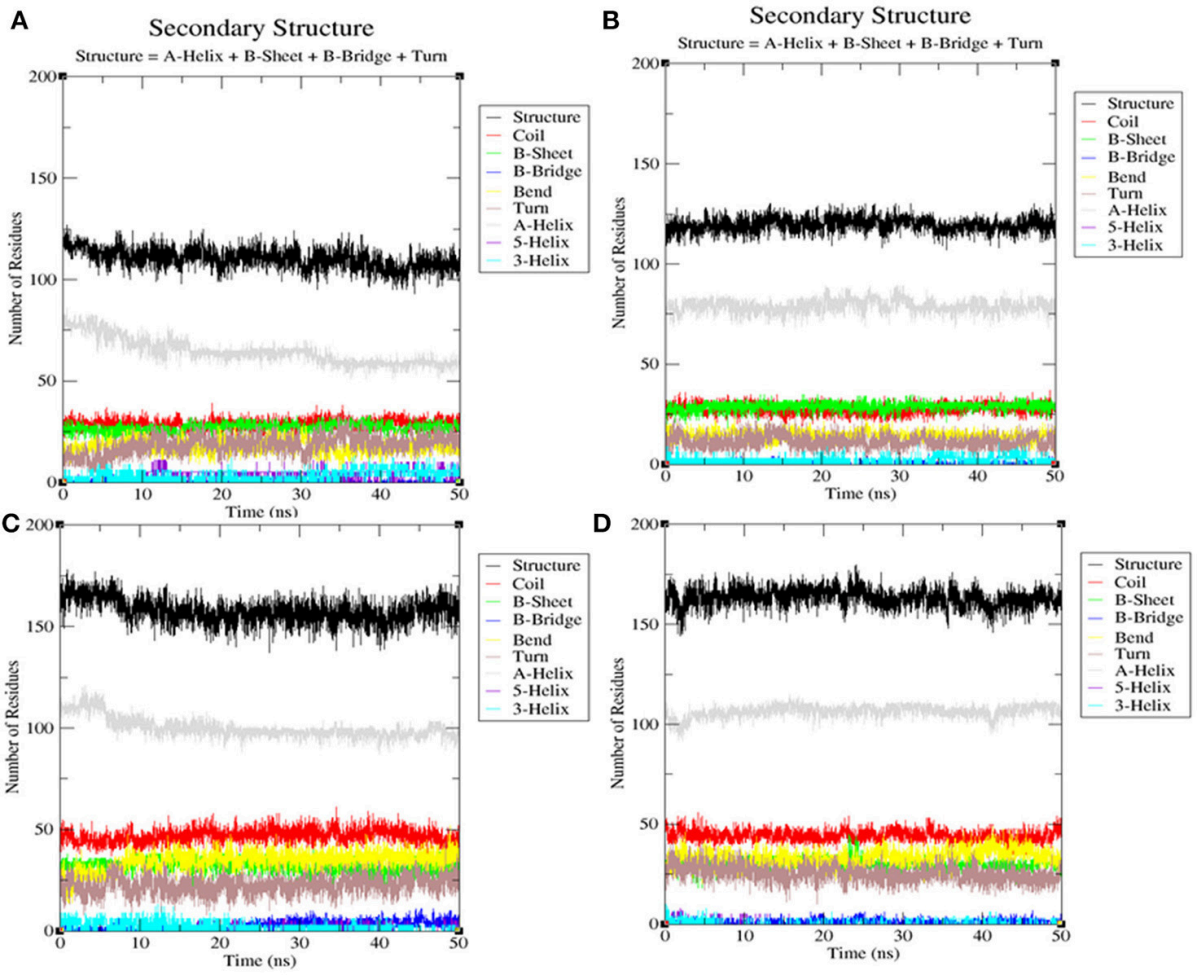
Secondary structure analysis indicating the structural elements of **(A)** LasR, **(B)** LasR**–**bakuchiol, **(C)** RhlR, and **(D)** RhlR**–**bakuchiol, respectively.

The secondary structures obtained during the MD simulation analysis are depicted in Figure 12. This analysis was aimed to measure the changes in the secondary structure of LasR and RhlR when bound with bakuchiol as a function of time. During the MD simulations, the secondary structure assignments such as α-helix, β-strand, and turns were broken into separate residues to measure the data in meaningful ways. The average number of residues contributing in the secondary structure formation was found to be more in the case of LasR–bakuchiol and RhlR–bakuchiol complexes than in LasR and RhlR, respectively (Table 7). This is due to the increase in α-helices in the protein structure. This analysis suggests that bakuchiol binding with LasR and RhlR leads to a considerable change in the secondary structure.

**Table 7:** Percentage of residues in LasR, LasR–bakuchiol, RhlR, and RhlR–bakuchiol that participated in average structure formation during 100 ns MD simulations*.

**Percentage of protein secondary structure (SS %)**								
**Protein type**	Structure*	**Coil**	β**-sheet**	β **-bridge**	**Bend**	**Turn**	α**-helix**	**3**_10_**-helix**
LasR	67	18	16	0	13	11	39	2
LasR-bakuchiol	73	16	18	0	9	7	49	1
RhlR	66	19	14	1	14	9	42	1
Rh1R-bakuchiol	68	18	13	1	14	11	44	0

* *Structure = α-helix + β-sheet + β-bridge + Turn*.

#### Hydrogen bond analysis

**Figure 13:**
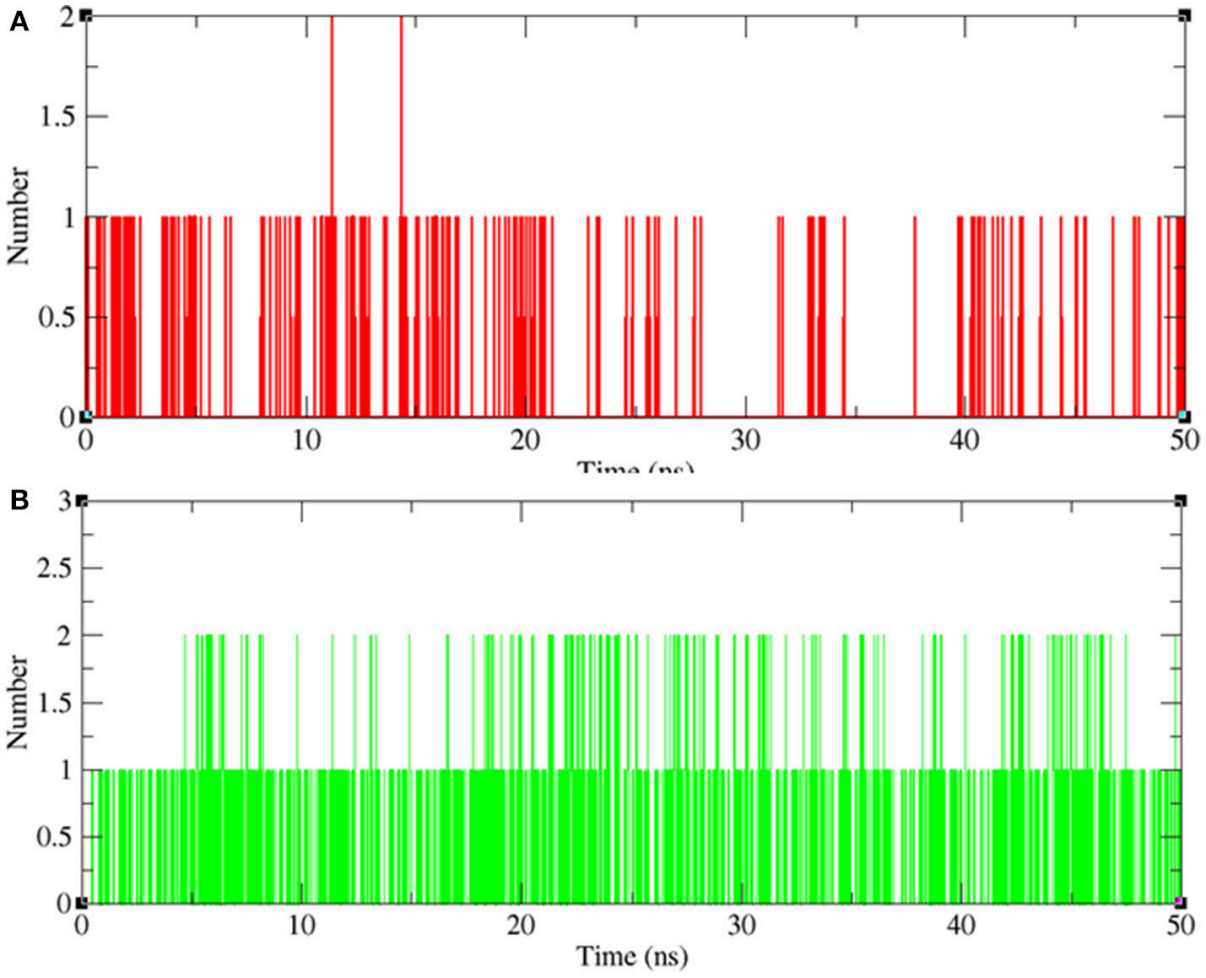
Hydrogen bond analysis between bakuchiol and **(A)** LasR (red) and **(B)** RhlR (green), respectively.

Hydrogen bonding between a receptor and ligands offers directionality and demonstrates the specificity of molecular interactions that are important aspects of molecular recognition (Hubbard and Kamran Haider, 2001). To validate the stability of docked complexes, the hydrogen bonds were paired within 0.35 nm between the protein and the ligands. During the 50 ns MD simulation studies for LasR-bakuchiol and RhlR-bakuchiol complexes, all calculations were performed in the solvent environment. Analysis revealed that bakuchiol binds to active pockets of LasR and RhlR with 1–2 hydrogen bonds (Figure 13).

## Conclusion

In conclusion, it is envisaged that the PCMF and bakuchiol obtained from *P. corylifolia* seeds may provide a possible substitute for the management of drug-resistant strains that cause infections/contamination, predominantly pathogens that form biofilms. The study highlights the anti-infective potential of the PCMF and bakuchiol instead of their bactericidal or bacteriostatic action, because the extract targets QS-controlled virulence and the biofilm. Computational analysis revealed that bakuchiol binds to the active pockets of LasR and RhlR during MD simulations. The binding of bakuchiol leads to structural deviations of LasR and Rh1R. This approach forms the basis of effective antimicrobial therapy in modern phytomedicine.

## Author contributions

IA, FH, MB, and FK designed and conceived experiments. FH, FK, MB, NA-S, AH, MR, MA, KL performed experiments. FH, FK, NA-S, AH, MR, MA, and KL analyzed and interpreted data. FH, IA, FK, MB, NA-S, AH, MR, MA, and KL wrote the manuscript and all the authors approved it.

### Conflict of interest statement

The authors declare that the research was conducted in the absence of any commercial or financial relationships that could be construed as a potential conflict of interest.
