# Supplementary material for: Seed Extract of Psoralea corylifolia and Its Constituent Bakuchiol Impairs AHL-Based Quorum Sensing and Biofilm Formation in Food- and Human-Related Pathogens
Source: Front Cell Infect Microbiol. 2018 Oct 25;8:351. doi: 10.3389/fcimb.2018.00351 (PMC6211212; doi:10.3389/fcimb.2018.00351)
Supplement: Supplementary file 1 [file Table_1.DOCX]

**Supplementary figures**

**Figure S1: Effect of respective ½ x sub-MICs of PCMF on growth of *A. hydrophila* (a) and *S. marcescens* (b)**
